# Correction: Bacterial actin MreB forms antiparallel double filaments

**DOI:** 10.7554/eLife.05394

**Published:** 2014-11-07

**Authors:** Fusinita van den Ent, Thierry Izoré, Tanmay AM Bharat, Christopher M Johnson, Jan Löwe

van den Ent F, Izoré T, Bharat TAM, Johnson CM, Löwe J. 2014. Bacterial actin MreB forms antiparallel double filaments. *eLife* **3**:e02634. doi: 10.7554/eLife.02634. Published 2 May 2014

In Table 2 in the published article, the headings for columns 6 and 7 were mistakenly combined, leading to all subsequent headings shifting one column to the left.

The article has now been corrected.

