# Supplementary material for: Bacterial actin MreB forms antiparallel double filaments
Source: eLife. 2014 May 2;3:e02634. doi: 10.7554/eLife.02634 (PMC4051119; doi:10.7554/eLife.02634)
Supplement: Supplementary file 1. — Plasmids used in this study. DOI: http://dx.doi.org/10.7554/eLife.02634.019 [file elife02634s001.docx]

| **Plasmid** | **Description** | **tag** | **Resistance** | **Promoter** | **Vector** | **Reference** |
| --- | --- | --- | --- | --- | --- | --- |
| pFE403 | *C. crescentus* MreB | His-SUMO* | kanamycin | T7 | pET28a | this work |
| CCM2 | *C. crescentus* MreB_ΔN-terminus | His | ampicillin | T7 | pHis17 | this work |
| pFE397 | *C. crescentus* MreB_ΔN-terminus _F102S/V103G | His | ampicillin | T7 | pHis17 | this work |
| CCM3 | *C. crescentus* MreB_ΔN- _F102S/V103G/S2834D | His | ampicillin | T7 | pHis17 | this work |
| pFE349 | *T. maritima* MreB | Intein-CBD* | ampicillin | T7 | pTXB1 | (Salje et al., 2011) |
| pFE52 | *T. maritima* MreB | His | ampicillin | T7 | pHis17 | (van den Ent et al., 2001) |
| pSENP1 | GST-SENP | GST | ampicillin | T7 | pGex6p1 | Kommander, pers comm. |
| pFB209 | *E. coli* MreB | No | ampicillin | Plac | pMLB1113 | (Bendezú and de Boer, 2008) |
| pFB112 | sdiA | No | tetracyclin | consititutive | pBR322 | (Bendezú and de Boer, 2008) |
| pFB124 | *E. coli* MreC, MreD-LE | No | spectinomycin | λP_R_ | pZC100 | (Bendezú and de Boer, 2008) |
| pFE535 | *E. coli* MreB_S284D | No | ampicillin | Plac | pMLB1113 | this work |
| pFE400 | *E. coli* MreB_V121E | No | ampicillin | Plac | pMLB1113 | this work |
| pFE496 | *E. coli* MreB_R124C/  C113S/C278S/C324S | No | ampicillin | Plac | pMLB1113 | this work |
| pFE491 | *E. coli* MreB_A125C/ C113S/C278S/C324S | No | ampicillin | Plac | pMLB1113 | this work |
| pFE493 | *E. coli* MreB_E262C/ C113S/C278S/C324S | No | ampicillin | Plac | pMLB1113 | this work |
| pFE494 | *E. coli* MreB_R188C/ C113S/C278S/C324S | No | ampicillin | Plac | pMLB1113 | this work |
| pFE489 | *E. coli* MreB_D78C/ C113S/C278S/C324S | No | ampicillin | Plac | pMLB1113 | this work |
| pFE500 | *E. coli* MreB_F84C/ C113S/C278S/C324S | No | ampicillin | Plac | pMLB1113 | this work |
| pFE502 | *E. coli* MreB_F84C/R124C/ C113S/C278S/C324S | No | ampicillin | Plac | pMLB1113 | this work |
| pFE497 | *E. coli* MreB_Q120C/ C113S/C278S/C324S | No | ampicillin | Plac | pMLB1113 | this work |
| pFE501 | *E. coli* MreB_F84C/Q120C/ C113S/C278S/C324S | No | ampicillin | Plac | pMLB1113 | this work |
| pFE499 | *E. coli* MreB_T119C/ C113S/C278S/C324S | No | ampicillin | Plac | pMLB1113 | this work |
| pFE488 | *E. coli* MreB_K77C/ C113S/C278S/C324S | No | ampicillin | Plac | pMLB1113 | this work |
| pFE503 | *E. coli* MreB_K77C/T119C/ C113S/C278S/C324S | No | ampicillin | Plac | pMLB1113 | this work |
| pFE498 | *E. coli* MreB_R122C/ C113S/C278S/C324S | No | ampicillin | Plac | pMLB1113 | this work |
| pFE485 | *E. coli* MreB_C113S/C278S/  C324S | No | ampicillin | Plac | pMLB1113 | this work |
| pFE520  pFE514  pFE505  pFE509 | *E. coli* MreB_C113S/C324S  *E. coli* MreB_E334C/ C113S/C278S/C324S/  *E. coli* MreB_Q29C/ C113S/C278S/C324S  *E. coli* MreB_E319C/ C113S/C278S/C324S | No  No  No  No | ampicillin  ampicillin  ampicillin  ampicillin | Plac  Plac  Plac  Plac | pMLB1113  pMLB1113  pMLB1113  pMLB1113 | this work  this work  this work  this work |
| Note that with the exception of pFB124 (a pSC101 derivative) all plasmids are derived of ColE1.  * Purification tag is cleaved off, leaving no extra residues on the protein of interest | | | | | | |
