# Supplementary material for: Bacterial actin MreB forms antiparallel double filaments
Source: eLife. 2014 May 2;3:e02634. doi: 10.7554/eLife.02634 (PMC4051119; doi:10.7554/eLife.02634)
Supplement: Supplementary file 2. — Crystallisation conditions. DOI: http://dx.doi.org/10.7554/eLife.02634.020 [file elife02634s002.docx]

**Table 2 – table supplement 1: Crystallisation data**

**d_e, drop 28b8**

0.1M Na acetate pH 4.5, 0.68 M NaH_2_PO_4_, 1.11 M Na_2_HPO_4_

cryo protectant: condition + 300 mM NaCl + 15 % glycerol

**s_2, drop 93h3**

0.2 M MES pH 6.0, 13.6 % (v/v) MPD

ADP nucleotide from protein prep

cryo protectant: condition + 300 mM NaCl + 25 % MPD

**s_2_a, drop 96f5**

0.14 M MES pH 6.0, 17.3 % (v/v) MDP

ADP nucleotide from protein prep, 1 mM A22 (in EtOH)

cryo protectant: condition + 300 mM NaCl + 25 % MPD

**s_2_m, drop 106f7**

0.14 M MES pH 6.0, 19.0 % (v/v) MPD

ADP nucleotide from protein prep, 1 mM MP265 (in H_2_O)

cryo protectant: condition + 300 mM NaCl + 25 % MPD

**s_e, drop 136a7**

0.01 M MES pH 6.5, 32 % (w/v) PEG 400

no cryo protectant added

**d_3, drop 144d6**

0.29 M MES pH 6.0, 9 % (v/v) MPD

5 mM AMPPNP / 10 mM MgCl_2_

cryo protectant: condition + 300 mM NaCl + 25 % MPD

**s_3_m, plate 235** (condition averaged since several crystals merged)

0.08 M Tris pH 8.5, 0.2 M MgCl_2_, 20 % (v/v) glycerol, 22.5 % (w/v) PEG 4000

5 mM AMPPNP / 10 mM MgCl_2_, 1 mM MP265 (in H_2_O)

cryo protectant: condition + 300 mM NaCl + 25 % glycerol

**m_2, drop 2447h1**

2 M NH_4_H_2_PO_4_, 0.01 % (w/v) PEG 3350

5 mM ADP / 10 mM MgCl_2_

cryo protectant: condition + 300 mM NaCl + 25 % glycerol

**m_3, 452a7**

0.1 M NH_4_H_2_PO_4_, 9.5 % (w/v) PEG 3350

5 mM AMPPNP / 10 mM MgCl_2_

cryo protectant: condition + 300 mM NaCl + 25 % PEG 200
